# Systemic immune-inflammatory biomarkers combined with the CRP-albumin-lymphocyte index predict surgical site infection following posterior lumbar spinal fusion: a retrospective study using machine learning

**DOI:** 10.3389/fmed.2025.1590248

**Published:** 2025-07-30

**Authors:** Zixiang Pang, Jiawei Liang, Jiayi Chen, Yangqin Ou, Qinmian Wu, Shengsheng Huang, Shengbin Huang, Yuanming Chen

**Affiliations:** ^1^Department Orthopedics Ward 3 (Spine and Osteopathy Surgery), Second Affiliated Hospital of Guangxi Medical University, Nanning, Guangxi, China; ^2^Department of Spinal and Bone Disease Surgery, Sixth Affiliated Hospital of Guangxi Medical University, Yulin, Guangxi, China; ^3^Department of Spine Surgery, Guigang City People’s Hospital, Guigang, Guangxi, China

**Keywords:** systemic immune-inflammation biomarkers, CRP-albumin-lymphocyte index, surgical site infection, machine learning, retrospective study

## Abstract

**Objectives:**

Emerging systemic immune-inflammatory biomarkers demonstrate potential for predicting postoperative complications. This study develops machine learning models to assess the combined predictive value of Aggregate Index of Systemic Inflammation (AISI), Systemic Immune-Inflammation Index (SII), CRP-Albumin-Lymphocyte (CALLY) index and Subcutaneous Lumbar Spine Index (SLSI) for surgical site infection (SSI) following posterior lumbar spinal fusion.

**Methods:**

This retrospective study analyzed 2,921 patients undergoing posterior lumbar spinal fusion at two tertiary hospitals in Guangxi (August 2017–January 2025). Data were partitioned into training (70%) and validation (30%) groups. Feature selection via univariate regression analysis identified predictive variables, followed by model development using ten machine learning algorithms: logistic regression (LR), support vector machine (SVM), random forest (RF), gradient boosting machine (GBM), XGBoost, neural network, K-nearest neighbors(KNN), AdaBoost, LightGBM, and CatBoost. Hyperparameters were optimized with 10-fold cross-validation. The top seven performing models (assessed by AUC, accuracy, sensitivity, specificity, precision, and F1 scores) were integrated into a dynamic nomogram. Internal validation employed ROC analysis and calibration curves, while Shapley Additive Explanations (SHAP) values interpreted feature importance in the optimal model.

**Results:**

Among 2,921 screened patients, 1,272 met inclusion criteria. Consensus feature selection across the seven top-performing ML algorithms identified AISI, SII, CALLY and SLSI as independent predictors of SSI. The derived nomogram demonstrated exceptional discrimination (training groups AUC = 0.966; C-index = 0.993, 95% CI 0.984–0.995) and excellent calibration. Additionally, the SHAP method emphasized the significance of AISI, SII, CALLY and SLSI as independent predictors influencing the machine learning model’s predictions.

**Conclusion:**

The AISI, SII, CALLY and SLSI emerged as independent predictors of SSI following posterior lumbar spinal fusion. Our machine learning-derived nomogram demonstrated high discriminative accuracy and clinical applicability through dynamic risk stratification. Leveraging the SHAP methodology enhances model interpretability, thereby empowering healthcare providers to proactively mitigate SSI occurrences and enhance overall patient outcomes.

## Introduction

Posterior lumbar spinal fusion stands out as a primary treatment option for lumbar degenerative diseases (LDD). Renowned for its consistent efficacy and minimal recurrence rates, this surgical technique has garnered significant popularity within the realm of spine surgery. Nevertheless, the incidence of surgical site infection (SSI) remains a prevalent complication following posterior lumbar spinal fusion, ranging from 0.2% to 16.1% (1). This complication is often associated with factors such as diabetes, obesity, increased blood loss, and prolonged surgical duration (2, 3). Recently, inflammation has emerged as a crucial phase in acute wound healing (4). Conversely, heightened activation of the proinflammatory cascade in patients preoperatively may predispose the host’s vulnerability to infection (5). The Aggregate Index of Systemic Inflammation (AISI), Systemic Immune-Inflammation Index (SII) and CRP-Albumin-Lymphocyte (CALLY) have emerged as innovative inflammatory biomarkers derived from immune cell subsets and platelet counts. These indices have been widely utilized to evaluate chronic inflammatory states and related diseases (6, 7). Nevertheless, to the best of our knowledge, there is a paucity of studies investigating the predictive impact of preoperative systemic immune inflammatory factors on the development of SSI following posterior lumbar spinal fusion. Hence, the integration of preoperative systemic immune-inflammation biomarkers with Subcutaneous Lumbar Spine Index (SLSI) for evaluating perioperative risk factors linked to SSI holds significant clinical relevance for spinal surgeons.

In the 21st century, artificial intelligence (AI) has been extensively applied and advanced in medical research, with the predictive capabilities of machine learning being widely acknowledged in the field of medicine (8). Machine learning (ML) is a potent data processing and computational tool that automatically filters and identifies key features to detect trends within data (9, 10). By analyzing extensive datasets, ML can pinpoint relevant clinical variables and predict target variables, thereby assisting medical researchers in effectively identifying the crucial factors influencing disease outcome (10).

In this study, univariate regression analysis was employed for initial feature screening, while a multi-machine learning algorithm was utilized to screen and validate the risk of SSI following posterior lumbar spinal fusion in conjunction with systemic immune-inflammatory factors and SLSI. The optimal clinical variables were identified through the convergence of these algorithms, leading to the development of a nomograms model for internal validation. Subsequently, Shapley Additive Explanations (SHAP) values were employed to discern and elucidate the predictive performance of variables within the model, thereby enhancing the interpretability and transparency of the model.

## Materials and methods

### Patients and study design

This study retrospectively examined patients with LDD who underwent posterior lumbar spinal fusion at the spine surgery departments of two tertiary level A hospitals in Guangxi between August 2017 and January 2025. The participating hospitals were the Second Affiliated Hospital of Guangxi Medical University, and Guangxi Guigang People’s Hospital. The inclusion criteria comprised the following: (1) Patients meeting clear indications for posterior lumbar interbody fusion (PLIF) and transforaminal lumbar interbody fusion (TLIF) surgeries. Exclusion criteria included: (1) Patients with a preoperative diagnosis of ankylosing spondylitis, spinal tumors, spinal infections, tuberculosis, or traumatic fractures; (2) Patients with uncontrolled infections in other body regions prior to surgery (such as severe pneumonia, severe urinary tract infections, intracranial infections); (3) Patients with a history of previous posterior lumbar fusion surgeries; (4) Operation time exceeded 5 h, and more than three lumbar levels were fused; (5) patients with severe gout or immune disorders; (6) Individuals with incomplete medical records, lost follow-up, or missing data.

**FIGURE 1:**
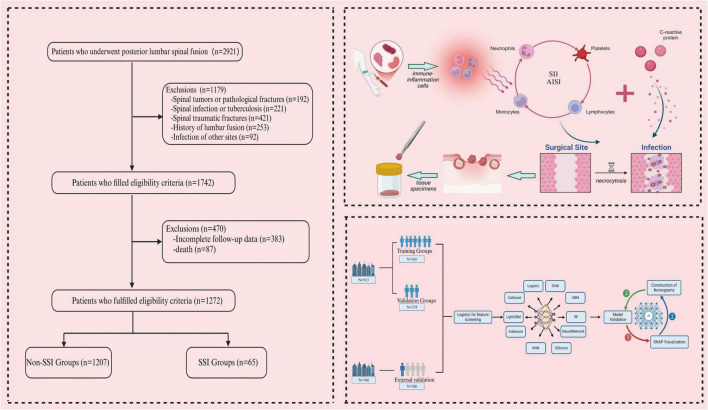
The patient flowchart and schematic diagram illustrating the mechanism of systemic immune-inflammatory biomarkers were created using BioRender.

Among the initial 2,921 patients screened, 1,649 individuals were excluded based on the predefined inclusion and exclusion criteria. Ultimately, our study comprised a total of 1,272 patients. The recruitment criteria for this retrospective study mandated that participating centers must be tertiary A hospitals with dedicated teams responsible for data collection to ensure the consistency and reliability of data entry. Furthermore, each participating center was required to contribute a minimum of 100 cases to establish a robust study cohort. All procedures were conducted by surgeons at the level of associate director or higher, each possessing over ten years of clinical surgical experience in their respective hospitals. To uphold data integrity, the staff involved in data collection verified data consistency through random sampling, followed by a meticulous review by the primary author to guarantee data quality. The patient flow chart and the diagram illustrating the inflammatory cell mechanism of action are presented in Figure 1. Subsequently, the data were randomly divided into a test group and a validation group for further analysis. Finally, patient data from Guigang People’s Hospital in Guangxi were utilized for external validation.

### Diagnostic criteria for SSI

The diagnostic criteria for SSI are as follows:(1) Redness, swelling, fever, and pain at the incision site were observed within 30–60 days postoperatively, accompanied by a significant increase in postoperative inflammatory markers; (2) Purulent secretions appeared at the operative mouth within 30–60 days after surgery and fever > 38°C (1); (3) Imaging studies, including CT, MRI, and ultrasound, revealed the presence of deep abscesses, abscess cavities, or tissue necrosis; (4) There was dehiscence or exudation at the incision site, and microbial culture results were positive; (5) Purulent exudate at the surgical site was identified within 30–60 days post-operation through a secondary surgical exploration, followed by microbial culture analysis to isolate the specific microorganisms (1, 11, 12).

Surgical site infection can be diagnosed when microorganisms are clearly cultured from surgical wound secretions. Additionally, SSI can be diagnosed if one of the criteria (2)–(4) is met, in conjunction with the criterion in (1).

### Definition of the systemic immune-inflammation biomarkers and CALLY

Laboratory tests were conducted within 24 h of admission, encompassing complete blood count, erythrocyte sedimentation rate (ESR), C-reactive protein (CRP) levels, albumin and coagulation function assessment. The AISI and SII are computed using the following formulas: SII = (neutrophil count × platelet count)/lymphocyte count; AISI = neutrophil count × platelet count × (monocyte count/lymphocyte count) (13, 14). The CALLY are computed using the following formulas: CALLY = albumin × lymphocyte count/CRP (15).

### Definition of subcutaneous lumbar spine index (SLSI)

The spinous process height (SPH) and subcutaneous fat thickness (SFT) were measured at the deepest point of the subcutaneous fat layer in the operative segment using preoperative sagittal T2-weighted MRI images. The formula for calculating the SLSI is as follows: SLSI = (SFT1/SPH1 + SFT2/SPH2… + SFT n/SPH n)/n (16).

### Data collection

The medical records of all hospitalized patients meeting the inclusion criteria were thoroughly reviewed and standardized. Based on a literature review and clinical experience, we identified 35 potential risk factors, including: (1) Demographic factors: gender, hypertension, diabetes, osteoporosis, rheumatism, smoking, alcohol, age, and body mass index (BMI); (2) Perioperative factors: subcutaneous lumbar spine index (SLSI) and blood loss; (3) Laboratory indicators: erythrocyte sedimentation rate (ESR), C-reactive protein (CRP), white blood cell (WBC), red blood cell (RBC), hemoglobin (HGB), hematocrit (HCT), mean corpuscular volume (MCV), mean platelet volume (MPV), platelet distribution width (PDW), eosinophils (EOS), basophils (BISO), plateletcrit (PCT), neutrophils (NEU), platelets (PLT), lymphocytes (LYM), monocytes (MONO), prothrombin time (PT), activated partial thromboplastin time (APTT), fibrinogen (FIB), thrombin time (TT), Systemic Immune-Inflammation Index (SII), Aggregate Index of Systemic Inflammation (AISI), CRP-albumin-lymphocyte (CALLY), and albumin.

### Data partitioning and validation

We randomly stratified 912 cases from the Second Affiliated Hospital of Guangxi Medical University in a 7:3 ratio, dividing the dataset into a training set (638 cases) and a validation set (274 cases). Of these, 70% of the data was used to train the model, while the remaining 30% was used for initial model validation. A separate external validation set was defined using 360 cases from Guigang People’s Hospital of Guangxi, which were not involved in model training or parameter tuning, and were only used for final performance testing.

The data partitioning process was carried out using the function from the package and the method from the package. A random seed was set to ensure reproducibility of the partitioning. During the stratified sampling, we used infection status (SSI/non-SSI) as the stratification variable, applied the function from the package, and set the hyperparameter to ensure the proportion of infections in the training, validation, and external validation sets remained similar.

**FIGURE 2:**
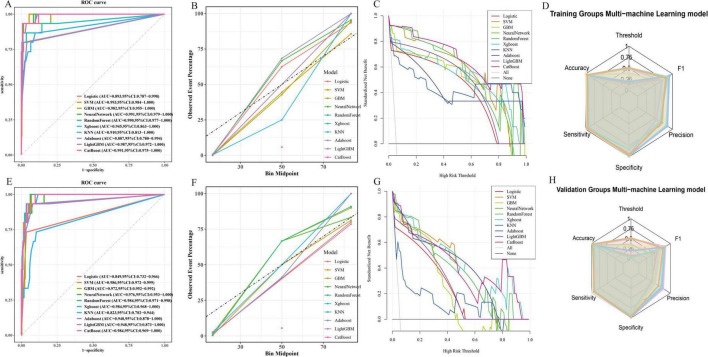
**(A)** Comparison of the AUC values of the 10 models in the training groups; **(B,C)** Fitting degree of the 10 models in the training groups; **(D)** Radar visualization of AUC values for 10 models in the training groups; **(E)** Comparison of the AUC values of the 10 models in the validation groups; **(F,G)** Fitting degree of the 10 models in the validation groups; **(H)** Radar visualization of AUC values for 10 models in the validation groups.

Subsequently, 10 machine learning algorithms were applied to train the datasets. The training process utilized Variance Inflation Factor (VIF), Recursive Feature Elimination (RFE), and regularization techniques, along with 10-fold cross-validation to optimize model hyperparameters and prevent overfitting (17). The convergence behavior of each model was examined using the loss function. At the end of each epoch, loss values were calculated and recorded. An early stopping mechanism was implemented to prevent overfitting, halting training when validation loss did not significantly decrease after a set number of iterations. Data preprocessing involved dataset expansion and removal of irrelevant hyperparameters. Model performance was evaluated using accuracy, sensitivity, specificity, precision, and F1 score. The performance of all machine learning models is shown in Figure 2.

### Statistical analysis

In this study, data analysis was conducted using IBM SPSS (version 23.0) and R Studio (version 4.4.1) for preliminary analysis and descriptive statistics of the dataset. Data following a normal distribution were presented as mean ± standard deviation, while data with a skewed distribution were expressed as median (quartile). The independent sample *t*-test was employed to compare differences in normally distributed data, while the Mann-Whitney U test was utilized for comparing non-normally distributed data. Frequency and percentage were used for categorical data, with inter-group differences assessed using chi-square tests. Model performance was evaluated by calculating Sensitivity and Specificity using receiver operating characteristic (ROC) curves, and calibration plots were used to assess model performance characteristics. Decision curve analysis (DCA) was conducted to evaluate the predictive ability of the model. Statistical significance in this study was defined as *p* < 0.05.

## Results

A total of 1,272 patients were included in this study. Data from 912 patients at the Second Affiliated Hospital of Guangxi Medical University were divided into a training group (638 patients, 39 with SSI, infection rate 6%) and a validation group (274 patients, 13 with SSI, infection rate 4%). Data from 360 patients at Guigang Peoples Hospital of Guangxi served as the external validation group (13 with SSI, infection rate 4%). Univariate logistic regression identified significant risk factors for SSI, including diabetes (*P* < 0.01), smoking (*P* = 0.043), alcohol (*P* = 0.022), BMI (*P* < 0.001), SLSI (*P* < 0.001) and various systemic immune-inflammation biomarkers such as, CRP, SII, and AISI (all *P* < 0.001) (Table 1). Multivariate analysis confirmed SII (*P* < 0.001), AISI (*P* < 0.01), and CALLY (*P* = 0.009) as independent predictors of SSI (Table 2).

**TABLE 1:** Baseline comparison and univariate analysis between patients in the non-surgical site infection (SSI) and SSI groups.

Variables	Non-SSI (*n* = 1,207)	SSI (*n* = 65)	t/χ^2^	OR	*P*-value
**Gender**					
Male	538 (44.6%)	33 (50.8%)	0.978	0.78	0.329
Female	669 (55.4%)	32 (49.2%)			
**Hypertension**					
No	851 (70.5%)	48 (73.8%)	0.576	0.85	0.565
Yes	356 (29.5%)	17 (26.2%)			
**Diabetes**					
No	932 (77.2%)	38 (58.5%)	−2.988	2.41	< 0.01**
Yes	275 (22.8%)	27 (41.5%)			
**Osteoporosis**					
No	347 (28.7%)	16 (24.6%)	−0.718	1.24	0.473
Yes	860 (71.3%)	49 (75.4%)			
**Rheumatism**					
No	974 (80.7%)	53 (81.5%)	−0.719	0.95	0.867
Yes	233 (19.3%)	12 (18.5%)			
**Smoking**					
No	758 (62.8%)	49 (75.4%)	2.263	0.55	0.043
Yes	449 (37.2%)	16 (24.6%)			
**Alcohol**					
No	796 (65.9%)	52 (80%)	2.711	0.48	0.022
Yes	411 (34.1%)	13 (20%)			
Age	62.6 ± 9.0	63.0 ± 8.2	−0.306	1.00	0.760
BMI	24.0 ± 3.1	26.5 ± 2.6	−6.354	26.37	< 0.001***
SLSI	0.5 ± 0.3	0.9 ± 0.3	−14.542	1.34	< 0.001***
Blood loss	432.8 ± 215.3	681.8 ± 192.2	−12.500	1.00	< 0.001***
ESR	14.2 ± 5.7	19.4 ± 6.4	−9.292	1.12	< 0.001***
CRP	8.6 ± 4.5	19.1 ± 4.9	−10.868	1.48	< 0.001***
WBC	8.7 ± 2.6	9.6 ± 2.4	−2.679	1.11	0.007
RBC	4.4 ± 0.7	4.4 ± 0.7	−0.131	1.02	0.895
HGB	125.2 ± 17.8	120.2 ± 22.9	1.759	0.99	0.029
HCT	11.2 ± 17.3	7.7 ± 15.0	1.804	0.99	0.116
MCV	86.7 ± 9.4	87.2 ± 8.3	−0.488	1.01	0.625
MPV	9.2 ± 1.1	9.2 ± 1.3	0.039	0.99	0.964
PDW	3.2 ± 4.8	2.3 ± 4.4	1.545	0.96	0.156
EOS	0.2 ± 0.1	0.2 ± 0.1	0.592	0.91	0.920
BISO	0.0 ± 0.0	0.0 ± 0.0	−0.486	74.63	0.502
PCT	0.2 ± 0.1	0.2 ± 0.1	0.639	0.29	0.523
NEU	5.1 ± 1.6	8.1 ± 0.9	−13.492	5.60	< 0.001***
PLT	270.6 ± 66.4	384.2 ± 28.5	−29.980	1.03	< 0.001***
LYM	2.0 ± 0.7	1.3 ± 0.4	9.020	0.07	< 0.001***
MONO	0.5 ± 0.2	0.8 ± 0.2	−10.808	12.98	< 0.001***
PT	11.3 ± 1.1	11.2 ± 0.8	0.407	0.95	0.684
APTT	28.7 ± 4.0	29.2 ± 3.9	−0.854	1.03	0.393
FIB	3.5 ± 0.9	3.4 ± 0.8	0.604	0.93	0.614
TT	15.0 ± 2.5	14.6 ± 2.2	1.190	0.93	0.231
Albumin	39.2 ± 5.2	28.8 ± 2.9	14.081	0.73	< 0.001***
SII	783.5 ± 440.9	2802.9 ± 1281.8	−16.408	1.00	< 0.001***
AISI	425.7 ± 314.7	1710.8 ± 696.8	−13.071	4.33	< 0.001***
CALLY	15.8 ± 17.4	3.5 ± 5.2	5.680	0.60	< 0.001***

“*P* < 0.05”, the representation was statistically significant; “**”, *P* < 0.01, indicates higher statistical significance; “***”, *P* < 0.001, indicates very high statistical significance. BMI, body mass index; SLSI, subcutaneous lumbar spine index; ESR, erythrocyte sedimentation rate; CRP, C-reactive protein; WBC, white blood cell; RBC, red blood cell; HGB, hemoglobin; HCT, hematocrit; MCV, mean corpuscular volume; MPV, mean platelet volume; PDW, platelet distribution width; EOS, eosinophils; BISO, basophils; PCT, plateletcrit; NEU, neutrophil; PLT, platelet; LYM, lymphocyte; MONO, monocyte; PT, prothrombin time; APTT, activated partial thromboplastin time; FIB, fibrinogen; TT, thrombin time; SII, Systemic Immune-Inflammation Index; AISI, Aggregate Index of Systemic Inflammation; CALLY, CRP-albumin-lymphocyte.

**TABLE 2:** Multivariate analysis of risk factors for surgical site infection (SSI).

Variables	β	OR	95% CI	*P*-value
Diabetes	1.435	0.92	0.08–10.50	0.947
Smoking	−2.116	0.10	0.96–1.98	0.215
Alcohol	−0.399	0.78	0.05–8.70	0.856
BMI	−0.403	1.06	0.43–1.09	0.120
SLSI	9.612	0.52	0.31–0.90	0.018
Blood loss	0.011	1.01	1.00–1.02	0.016
ESR	0.257	1.16	0.94–1.44	0.175
CRP	0152	2.20	1.39–3.49	< 0.001***
WBC	−0.057	1.55	0.34–1.89	0.150
HGB	0.007	0.98	0.92–1.04	0.465
NEU	−0.844	2.50	0.41–15.21	0.321
PLT	0.044	1.08	1.01–1.15	0.020
LYM	1.825	0.02	0.30–7.66	0.196
MONO	13.341	1.56	0.14–2.68	0.209
Albumin	0.236	0.47	0.23–0.93	0.031
SII	0.008	1.00	1.00–1.01	< 0.001***
AISI	−1.211	0.36	0.15–6.06	< 0.01**
CALLY	0.297	1.47	1.10–1.96	0.009

“*P* < 0.05”, the representation was statistically significant; “**”, *P* < 0.01, indicates higher statistical significance;“***”, *P* < 0.001, indicates very high statistical significance. BMI, body mass index; SLSI, subcutaneous lumbar spine index; ESR, erythrocyte sedimentation rate; CRP, C-reactive protein; WBC, white blood cell; HGB, hemoglobin; NEU, neutrophil; PLT, platelet; LYM, lymphocyte; MONO, monocyte; SII, Systemic Immune-Inflammation Index; AISI, Aggregate Index of Systemic Inflammation; CALLY, CRP-albumin-lymphocyte.

### Best evaluation of machine learning models

We evaluated 10 machine learning models to guide clinical management and found that the SVM model performed best. Specifically, for the training group, the accuracy = 0.965, sensitivity = 0.963, specificity = 0.982, precision = 0.865, and F1 score = 0.865. For the validation group, the accuracy = 0.951, sensitivity = 0.981, specificity = 0.986, precision = 0.778, and F1 score = 0.782. The SVM model is capable of addressing non-linear problems and achieving complex data classification through high-dimensional mapping by selecting appropriate kernel functions. Consequently, SVM outperforms traditional linear classifiers when dealing with complex, non-linearly separable datasets (10, 18). This study initially considered 35 risk factors potentially affecting SSI. Given the diversity of indicators in this study, both linear and non-linear relationships exist. After eliminating irrelevant factors using 10-fold cross-validation and RFE iteration during the SVM training process, the model identified four independent risk factors associated with SSI: SII, AISI, CALLY, and SLSI.

### SHAP-based model interpretability analysis

In this study, the SHAP method was utilized to identify the most influential clinical features in the machine learning model. Shapley values quantified each variable’s average contribution across all possible combinations (19). Through recursive feature elimination, less impactful features were iteratively removed, and the model was re-fitted until a significant decline in performance was observed (20, 21).

**FIGURE 3:**
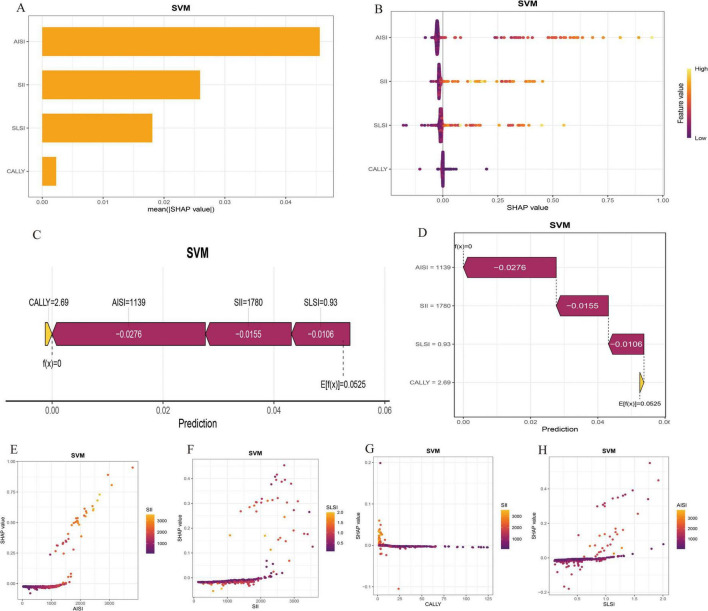
Shapley Additive Explanations (SHAP) interpretation of key hazard features in the optimal support vector machine (SVM) model. **(A)** Risk factor characteristic importance weight display. **(B)** The final selected clinical features’ contribution to predicting individual independence in the SSI model is illustrated. Each point’s position indicates the feature’s impact on risk prediction, with color denoting the feature’s predictive value. **(C,D)** Personalized patient predictions. Higher salience is indicated by longer bars in the chart. **(E–H)** The SHAP dependency plot illustrates how a single feature influences the prediction model output, with each data point representing a prediction from an individual patient.

Within the SVM framework, SHAP values were employed to interpret the interactions between systemic immune-inflammation biomarkers, CALLY and SLSI. Figure 3A illustrates the positive predictive roles of AISI, SII, CALLY, and SLSI in SSI. Figure 3B displays sample-specific feature importance, using a color gradient to indicate eigenvalue magnitude, while the vertical axis ranks feature importance. For individual sample interpretability, Figures 3C, D visualize SHAP values, elucidating feature interactions influencing SSI occurrence. Figures 3E–H demonstrate the correlations and dependencies among the factors. This analysis assists in developing targeted prevention and management strategies.

### Model development

**FIGURE 4:**
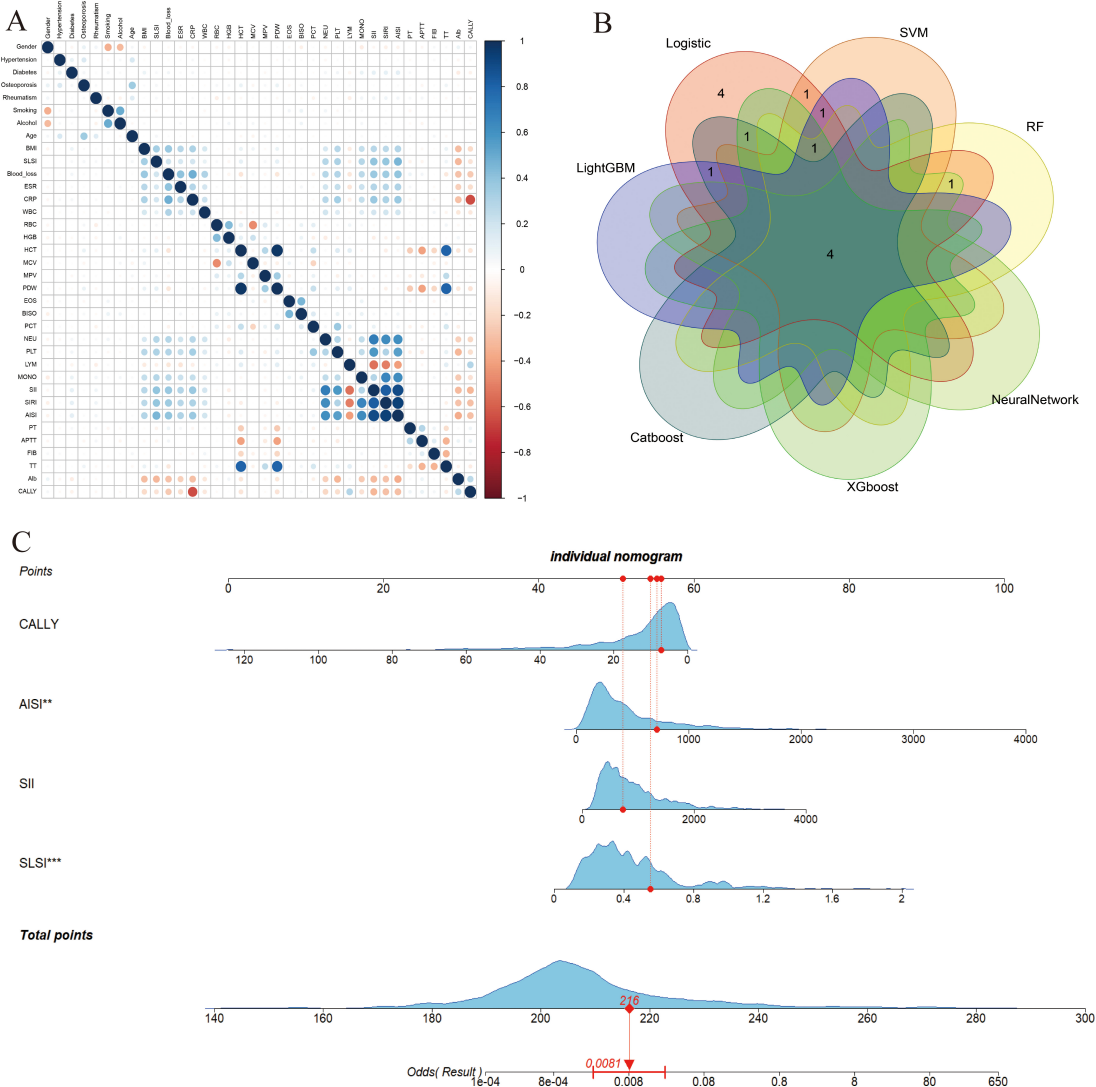
**(A)** Heat map of the correlations between all the variables are shown; **(B)** The intersection of variables screened using seven machine learning methods; **(C)** Visualization nomogram model for surgical site infection (SSI) after posterior lumbar spinal fusion. “**”, indicates a higher level of statistical significance; “***”, indicates very high statistical significance. SII, Systemic Immune-Inflammation Index; AISI, Aggregate Index of Systemic Inflammation; CALLY, CRP-albumin-lymphocyte index; SLSI, Subcutaneous Lumbar Spine Index.

**FIGURE 5:**
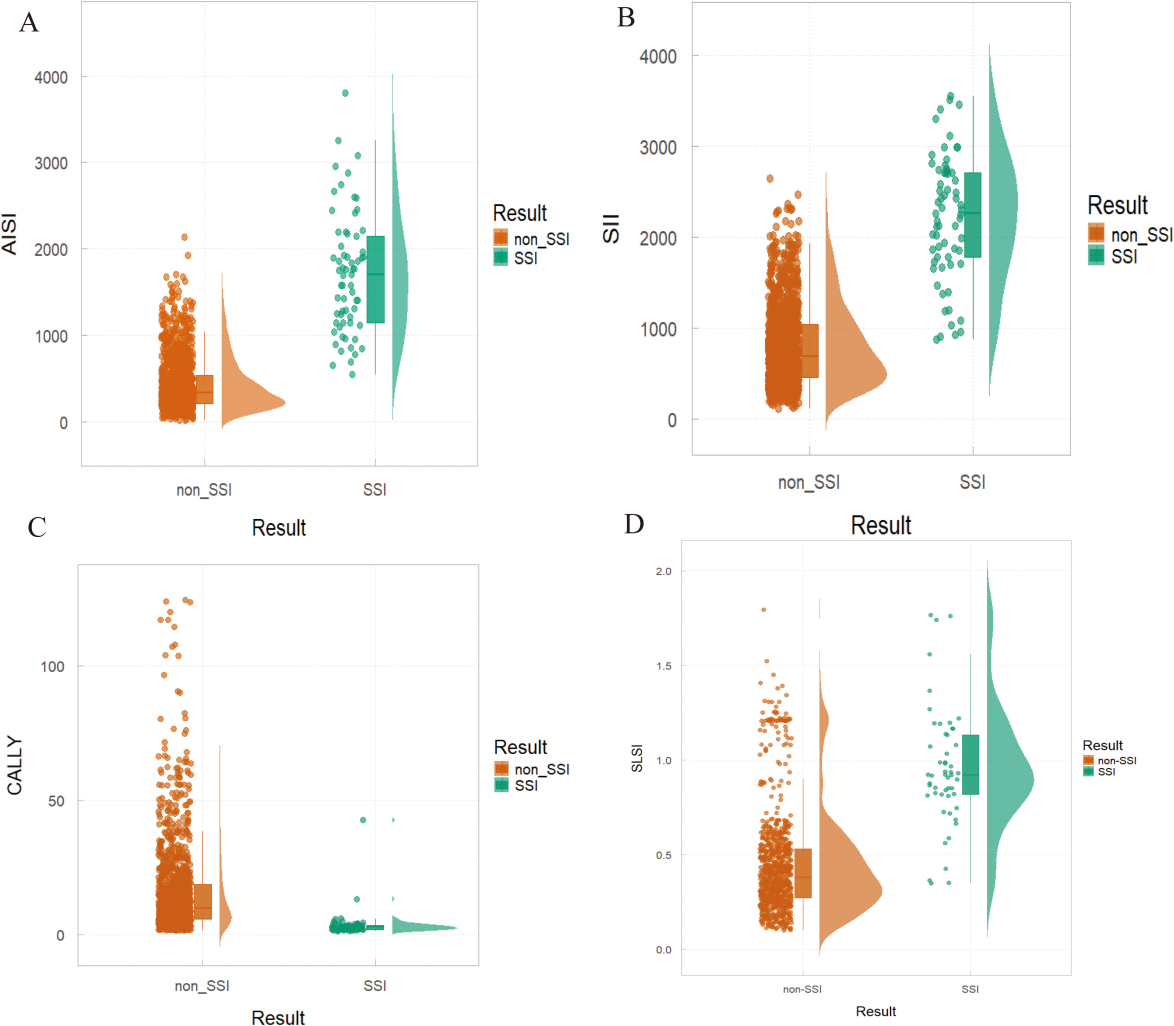
Differences in risk factors between non-surgical site infection (SSI) and SSI groups. **(A)** AISI: *P* < 0.01; **(B)** SII: *P* < 0.001; **(C)** CALLY: *P* = 0.009; **(D)** SLSI: *P* = 0.018.

The Venn diagram (Figures 4A, B) visually summarizes the final risk factors identified by the top seven machine learning models, leading to the selection of four independent predictors: AISI, SII, CALLY and SLSI. Dynamic nomograms were developed based on these variables (Figure 4C). A comparative analysis revealed significant intergroup differences in systemic immunoinflammatory biomarkers (AISI, SII), CALLY, and SLSI between the SSI and non-SSI groups (Figure 5).

### Model performance and evaluation

**FIGURE 6:**
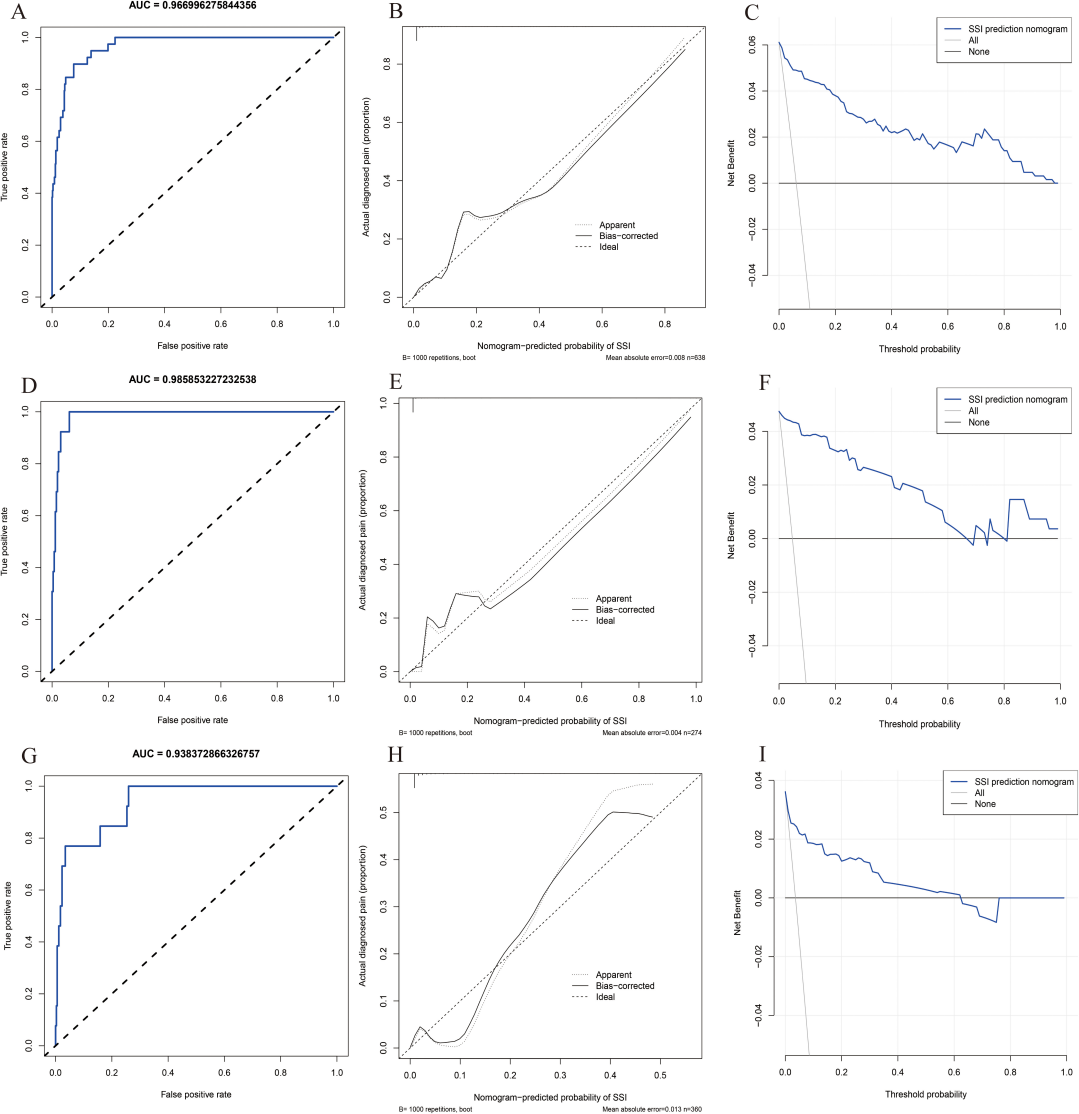
Model performance and evaluation. **(A–C)** Receiver operating characteristic (ROC), decision curve analysis (DCA) and calibration curve for the training model; **(D–F)** ROC, DCA and calibration curve for the validation model; **(G–I)** ROC, DCA and calibration curve for the external validation model.

Model performance was assessed using ROC and calibration curves, with an AUC of 0.966 in the training groups, indicating high predictive accuracy. The calibration curve showed strong agreement between predicted and actual values, and the model’s C-index was 0.993 (95% CI 0.984–0.995), reflecting excellent discrimination. The validation groups yielded an AUC of 0.985 and a C-index of 0.986 (95% CI 0.972–0.999). The external validation groups achieved an AUC of 0.938, with calibration and decision curves demonstrating strong consistency with the training and internal validation groups (Figure 6).

## Discussion

Surgical site infection is a common and serious complication following posterior lumbar spinal fusion (2, 3). While numerous studies have investigated the pathogenesis of SSI in this context, there is a paucity of literature focusing on predicting SSI through the lens of systemic immune-inflammatory factors using machine learning techniques. Recent research has examined the screening and diagnosis of early SSI through laboratory blood inflammatory markers, such as CRP, ESR and their individual derivatives, including lymphocyte, platelet, and neutrophil counts (5, 22). In this study, we introduced a range of compound blood inflammatory markers, including the SII, AISI and CALLY, in combination with the SLSI. This study comprehensive analysis aims to elucidate the mechanisms of action and the clinical significance of systemic blood inflammatory immunity and lumbar fusion segment-specific local surgical index in the occurrence of SSI.

In SSI pathogenesis, heightened oxygen demand at incision sites initiates a pathological cascade characterized by inflammatory dysregulation. This process perpetuates microvascular dysfunction through impaired perfusion and structural damage to capillary networks, establishing a self-sustaining cycle of tissue compromise (23, 24).

Monocytes are crucial in regulating inflammation, with elevated levels indicating damage to the oxidative respiratory chain, which can increase blood glucose and oxygen consumption (25, 26). Lymphocyte count before surgery can predict susceptibility to infections, as lymphocytes self-destruct and release anti-inflammatory cytokines to regulate inflammation and maintain immune balance (27, 28). Platelets accumulate early at injury sites, coordinating both acute and chronic inflammation, releasing soluble factors, interacting with immune cells, and promoting tissue repair while influencing fibrosis (29). Neutrophils are central to acute inflammation, secreting cytokines and enzymes like metalloproteinase 9 and vascular endothelial growth factor to aid matrix remodeling and immune responses (25). Dysfunctional neutrophils can cause capillary obstruction, impair pathogen clearance, and hinder healing, increasing infection risk (27, 30). Albumin, a key biomarker of nutritional status and immune function, inversely correlates with inflammation and maintains antioxidant properties. The CALLY index, combining immunoinflammatory and nutritional markers, is a sensitive prognostic tool for cancer and surgical outcomes (31, 32).

Single peripheral blood immunoinflammatory markers exhibit significant variability and limited predictability for forecasting SSI. In contrast, composite indices, such as the SII, AISI, and CALLY index, which incorporate multiple serum immunoinflammatory markers, offer a more comprehensive assessment. Preoperative inflammatory cascades trigger alterations in various peripheral blood markers, resulting in elevated systemic immune-inflammatory biomarker levels that indicate increased overall inflammation. This, in turn, enhances predictive accuracy. Moreover, composite indices help reduce false positives and the uncertainty associated with relying on individual inflammatory factors for SSI prediction (33). Patients with elevated SII and AISI levels signify a pronounced preoperative inflammatory state, warranting extended postoperative low-grade antibiotic administration and prompt management of postoperative blood glucose levels. Preoperative potassium permanganate immersion can effectively reduce residual bacteria and microorganisms in skin folds. Conversely, patients with low CALLY levels necessitate meticulous regulation of albumin levels during the perioperative phase, preoperative protein supplementation, postoperative nutritional support in collaboration with the nutrition department, and adherence to a high-protein diet.

Prior studies have primarily utilized BMI for SSI assessment, yet their reliability is hindered by variations in muscle mass and fat infiltration distribution at the fusion site (34, 35). In posterior lumbar fusion, an elevated SLSI signifies increased subcutaneous fat or a shorter spinous process, complicating surgical exposure. Extended intraoperative muscle traction can disrupt muscle fiber integrity in the surgical region, heightening the likelihood of muscle dysfunction (35, 36). Higher SLSI levels necessitate more extensive soft tissue removal with electrotome, elevating postoperative fat liquefaction risk. Patients with elevated SLSI levels may undergo deeper incisions, leading to increased residual space within the surgical cavity, thereby enhancing the accumulation and proliferation of local pathogenic microorganisms (16). In routine clinical practice, it is crucial to tailor individualized surgical strategies for patients with elevated SLSI levels. For instance, spinal surgeons can opt for preoperative decompression of the canalis spinalis and nerve root canals using minimally invasive spinal endoscopy. This method reduces soft tissue trauma, maintains spinal stability with percutaneous pedicle screw placement, minimizes muscle traction, and restricts the use of electrotome. Additionally, preserving the integrity of the deep intermuscular fascia and employing prolonged irrigation with a saline solution during surgery can help reduce residual microorganisms, thereby lowering the risk of surgical site infections.

Support vector machine improves upon traditional linear regression by using a kernel function to map non-linear data to a higher-dimensional space, making it linearly separable. SVM builds the decision boundary using support vectors, which are the critical data points closest to the boundary and directly influence the hyperplanes position (18, 37). This mechanism reduces the need for all data points, making SVM robust, especially in high-dimensional data. By minimizing structural risk, SVM minimizes generalization error and model complexity, improving its ability to generalize. This makes SVM a valuable tool in predicting SSI and enhancing clinical decision support systems (18).

Shapley Additive Explanations, based on game theory, helps interpret machine learning models by calculating the contribution of each feature to predictions (21). The SHAP Summary Plot shows the overall impact of features like SII, AISI, CALLY, and SLSI on model predictions, with higher values indicating a greater influence on SSI occurrence. The SHAP Force Plot illustrates individual feature contributions to a single prediction. The SHAP Waterfall Plot clarifies feature impact on predictions and the prediction path. The SHAP Dependence Plot reveals both individual feature effects and interactions with others. Using SHAP, we systematically predicted independent risk factors for SSI, visualizing feature impacts in both global and local contexts.

In this study, we developed the optimal SVM model using machine learning and identified the final independent factors by integrating SHAP with a nomogram. Our results indicated that AISI demonstrated the highest predictive capability among the systemic inflammatory biomarkers evaluated. This is due to its ability to comprehensively analyze neutrophils, platelets, monocytes, and lymphocytes (13, 14). Compared to SII and CALLY, AISI more accurately reflects the onset, progression, and prognosis of the inflammatory cascade prior to surgery. Furthermore, the SLSI in our nomogram model exhibited strong predictive value, as it more effectively assessed the overall local condition of the surgical site before the operation. The comprehensive evaluation of muscle, fat, and spinal structures enables surgeons to better anticipate the likelihood of SSI, providing them with greater psychological preparedness.

It is important to acknowledge the limitations of this study. Although data were collected from two Grade-A tertiary hospitals in Guangxi, and efforts were made to minimize errors and biases, variations in surgeon proficiency, surgical volume, and technique preferences may have influenced SSI outcomes. The study population may not fully represent the general population, and its retrospective design introduces potential recall bias. Additionally, unmeasured inflammatory markers, such as preoperative procalcitonin and interleukin-6, could limit causal inference. The post-surgery dynamics of SII, AISI, and CALLY were not examined, and the study considered only a limited range of surgical risk factors. Future research should expand the dataset to improve model performance. Furthermore, patients in the SSI group had higher systemic inflammatory biomarker levels and SLSI scores compared to those in the non-SSI group. This suggests that patients with SSI generally had poorer overall health and were more susceptible to related complications. Despite these findings, the results indicate that SII, AISI, CALLY, and SLSI can effectively distinguish between SSI and non-SSI patients. Therefore, larger-scale prospective studies across multiple centers are needed to validate these conclusions.

## Conclusion

The findings of this study indicate that the SII, AISI, CALLY and SLSI can effectively predict adverse outcomes related to SSI in patients undergoing posterior lumbar spinal fusion. Furthermore, our model demonstrates well predictive power and clinical applicability.

## Data Availability

The raw data supporting the conclusions of this article will be made available by the authors, without undue reservation.
